# Evaluation of the interval cancer rate and its determinants on the Girona health region’s early breast cancer detection program

**DOI:** 10.1186/1471-2407-14-558

**Published:** 2014-08-01

**Authors:** Gemma Renart-Vicens, Montserrat Puig-Vives, Joan Albanell, Francesc Castañer, Joana Ferrer, Miquel Carreras, Joan Tarradas, Maria Sala, Rafael Marcos-Gragera

**Affiliations:** Research Group on Statistics, Applied Economics and Health (GRECS), CIBER of Epidemiology and Public Health (CIBERESP), University of Girona, Campus de Montilivi, 17071 Girona, Spain; Oncology Director Plan, Health Department, Epidemiology Unit and Girona CancerRegistry (UERCG), Girona, Spain; Research Group on Statistics, Applied Economics and Health (GRECS), CIBER of Epidemiology and Public Health (CIBERESP), Girona Biomedical Research Institute (IdiBGi), Girona, Spain; Hospital Sta. Caterina, Salt, Spain; Institut d’Assistència Sanitaria, Girona, Spain; Hospital de Palamós, Palamos, Spain; Servei d’Epidemiologia i Avaluació. Institut Hospital del Mar d’Investigacions Mèdiques (IMIM), Barcelona Red de Investigación en Servicios Sanitarios en enfermedades crónicas (REDISSEC), Barcelona, Spain; Epidemiology Unit and Girona Cancer Registry (UERCG), Oncology Director Plan, Health Department, Girona Biomedical Research Institute (IdiBGi), Girona, Spain

**Keywords:** Interval cancer rate, Propotional incidence, Clinicopathological characteristics

## Abstract

**Background:**

The main aim of this study is to estimate the rate of false negative and true IC on the Program for the Early Detection of Breast Cancer (PEDBC) run by the Girona Health Region (GHR) and compare the clinicopathological characteristics of these tumors with those detected within the same program.

**Methods:**

A retrospective cohort study including all women participating on the Girona PEDBC between 2000 and 2006, with negative mammography screening. The IC included are those detected between the first and second round of screening and between the second and third round.

**Results:**

We identified a total of 43 IC, representing an incidence rate of 0.70 cases per 1,000 screened women. Of the 43 probable IC, we were able to classify a total of 22 (51.2%) cases. Of these 22 cases, 54.5% were classified as true interval tumors, 13.6% false negatives, 18.2% occult tumors and the remaining 13.6% minimal sign.

We found significant differences in some clinicopathological characteristics of the IC comparing with the tumors detected within the program during the same period.

**Conclusions:**

The IC rate for the PEDBC is within the expected parameters, with a high proportion of cases of true interval cancers (54.5%) and a low proportion of false negatives (13.6%). The results show that the proportional incidence of IC is within the limits set by European Guidelines. Furthermore, it has been confirmed that IC display more aggressive clinicopathological characteristics than screening breast cancers.

## Background

Breast cancer is the most common cancer in Spanish women. In Spain, approximately 16,000 cases are diagnosed and 6,000 deaths occur annually due to this disease [1]. Breast cancer mortality in Western countries has followed a downward trend since the early 90s [2]. It has been estimated that the use of screening mammography and adjuvant treatments for breast cancer have had a similar impact on improving survival [3].

The natural history of breast cancer, with its long preclinical phase, favors the possibility of early detection through mammography screening. The introduction of screening programs for breast cancer have reduced mortality from this neoplasm between 10% and 35% [3–5], varying by age, years of follow-up, number of women screened and frequency of mammography. However, certain adverse effects of mammography screening have to be considered. The most important are interval cancer and false negative breast cancers. Analysis of inteval cancers (IC) is critical in determining screening sensitivity and represents an objective measure of the quality of the screening program in the sense that increased detection of tumors on the program must lead to a lower incidence of IC. So the interval cancer rate is a key component of quality control for programs using both conventional and digital mammography.

The IC, as defined by the European Guidelines for Quality Assurance in the Screening and Diagnosis of Breast Cancer [6], is a primary breast tumor diagnosed in a woman who has undergone screening, with or without additional assessment, and the result was negative for malignancy. The diagnosis must be made before the next invitation onto the program or within a period equal to the screening interval if the woman has reached the age limit for participation. Published studies [7–9] show that IC and screen-detected tumors have different clinicopathologic characteristics, IC being more aggressive. IC tends to have a worse prognosis, with a higher proportion of large tumors, lymph node involvement, advanced stages, high histologic grade and negative hormone receptors.

However, IC tumors are a heterogeneous group of tumors. It can be classificate into four categories by the retrospective review of both screening and diagnostic mammograms: true interval cancers, false-negative cancers, minimal-signs and occult tumors. True interval cancers are those that showed normal or benign features in the previous screening mammogram; false-negative cancers are detected when signs suspicious for malignancy are retrospectively seen on a mammogram; minimal-signs are cancers showing detectable but non-specific signs at the latest screening; and occult tumors are those that present clinical signs of the disease despite a lack of mammographic abnormalities either at screening or at diagnosis.

Information on IC and the false-negative, both related to women and to program, is useful for assessing and adapting screening strategies, for evaluating the work of radiologistsand thereby reducing the proportion of false negatives, achieving higher screening sensitivity.

Although IC are inevitable in a screening program, it is recommended that their frequency should kept very small, since a high proportion would decrease screening effectiveness. In Europe, several studies have assessed IC within the framework of screening programs [10–16]. In general, reported incidents do not exceed the limits recommended by European Guidelines (incidence <0.30 the first year and <0.50 the second).

The main aim of this study is to estimate the rate of false negative and true IC on the Program for the Early Detection of Breast Cancer (PEDBC) and compare the clinicopathological characteristics of these tumors with those detected within program.

## Methods

### Design and study population

We performed a retrospective study including all women screened in the Girona PEDBC between 1 January 2000 and 31 December 2006, and followed up until June 2009 with a negative mammography screening; 32,783 women.

The study period involves the IC detected between the first and second round of screening and between the second and third round. We included both invasive (ICD-O-3: C50.0-C50.9) and *in situ* tumors (ICD-O-3: D050-D059) [17] and for simultaneous bilateral tumors, the most aggressive of the two was considered.

All women resident in Girona Health Region aged between 50 and 69 years are actively invited to participate in the propulation-based screening program every 2 years. The Girona Health Region’s (GHR) PEDBC was introduced with a pilot testing in 1999 and was extended throughout the GHR in 2001. Following the European guideline recommendations [6], the test performed is the double projection mammography and double reading every two years. During the study period, the PEDBC consisted of six radiological units covering aproximately 20% of the female population in 2006 and the participation rate was around 64%. Only one of these units switched to digital mammography in 2004.

The Girona Cancer Registry (GCR) is a population-based registry that collects information on all cases of breast and female genital cancer diagnosed in patients living in the province of Girona since 1980, expanded to all tumor sites since 1994. According to the 2007 census, the GCR covered a population of 339,660 women, representing 9.4% of the Catalan population. Additionally, during the period 2007-09, the quality data indicators of the GCRwere as follow: proportion of death certificate only (DCO) of 2.7%, the histological verification (VH) of 91.2% and a mortality-incidence ratio (M/I) of 30.2%.

Study data were collected using a protocol approved by the ethics committes of the University Hospital “Doctor Josep Trueta” (CEIC-Hospital Josep Trueta), Girona. Specific patient consent was not requiered because we used retrospective data from screening participants who had previously signed information release documents.

To identify probable IC, the PEDBC and GCR databases have been cross-referenced. From these databases, information has been collected from all women who participated at least once on the program between 01/01/2000 and 31/12/2006. However, in order to ensure follow-up for all the women screened within the study period, the GCR has provided population data for women with breast tumors between 01/01/2000 and 30/06/2009.

After identifying probable cases of IC, the last mammography screening and diagnostic mammography for breast cancer was recovered for each case. A panel of expert radiologists who regularly interpret mammograms in the programme, classified the IC into true intervals, false negatives, occult tumors and minimal signs following the agreed protocol. It consisted on reviewing both screening and diagnostic mammograms through independent double reading with arbitration. First, the radiologists reviewed the screening mammograms without seeing the diagnostic mammogram and classified into positive (abnormality clearly visible and warrants assessment), negative (normal mammogram), and minimal-signs (subtle abnormality, not necessarily regarded as warranting assessment). Afterwards, the radiologists reviewed together the diagnostic and screening mammograms and classified into true interval cancers, false negatives, minimal-signs cancers and occult tumors.

In turn, tumors detected by the PEDBC during the study period have been identified. The following clinicopathological characteristics of screening tumors and interval cancers were collected: age, stage (0-IV), tumor size (≤9, 10-14, 15-19, 20-29, 30-49, ≥50 cm), number of positive lymph nodes (none, 1-3, >3), histological grade (poor, moderate, good), histology (invasive, *in situ*) hormone receptor status (estrogen and progesterone receptors), HER2 (human epidermal growth factor receptor 2) and molecular subtype (luminal A, luminal B, HER2-overexpressed, triple negative) [18].

### Analysis

The IC rate has been estimated as the number of tumors diagnosed in a defined time period since the last negative screening examination for every 1,000 women with negative mammography screenings. Confidence intervals for the incidence rates of IC have been estimed assuming a Poisson distribution.

Proportional incidence was estimated as the ratio of the observed incidence of IC compared to the baseline incidence expected in the absence of screening. The baseline incidence rates were estimated using the incidence in the 50-59 and 60-69 years old age groups in the period before screening, 1980-1989. A generalized linear model with poisson distribution was used to estimate and projecte on to the following years to obtain the incidences rates per 10,000 women. In the 50-59 and 60-69 years old age groups, respectively, these were 13.77 and 22.2 for first round, and 13.93 and 22.82 for the second.

We also calculated the sensitivity of the screening test, which, according to the European Guidelines definition, is the ability to identify a case during its detectable phase, it being advisable to estimate it as the number of cases detected by screening from the total number of tumors detected in screened women (tumors detected by the PEDBC and IC).

These indicators were stratified by age group (50-59 and 60-69), type of screening (initial and subsequent), round number on the program and time elapsed between last mammography screening and diagnosis (less than 12 months, between 12-24 months). To compare clinical characteristics between cases detected by the screening program and the IC, the Chi-squared statistical test was used. The statistical analysis was performed using version 19.0 of the SPSS.

## Results

Table 1 shows the incidence rate for the IC and tumors detected on the program during the study period, as well as the classification of these probable IC. We identified a total of 43 IC, representing an incidence rate of 0.70 cases per 1,000 screened women. During the same period, 299 tumors were detected on the PEDBC, representing a detection rate of 4.9 tumors per 1,000 screened women.

**Table 1:** **IC and PDPCM cancer incidence rate and IC classification**

	N (‰)	N (%)
**Screening tumors [n (rate)]**	299 (4.9)	
**Interval tumors [n (rate)]**	43 (0.70)	
Classified		22 (51.2)
True interval		12 (54.5)
False Negative		3 (13.6)
Occult Tumors		4 (18.2)
Minimal Signs		3 (13.6)

Of the 43 probable IC, we were able to classify a total of 22 (51.2%) cases. Of these 22 cases, 54.5% were classified as true interval tumors, 13.6% false negatives, 18.2% occult tumors and the remaining 13.6% minimal sign.

Table 2 shows the incidence rate of the IC according to age group, screening type and program round. Of the 43 IC detected, 30 (69.8%) occurred in women aged 50 to 59 and 13 (30.2%) in women aged 60 to 69. Furthermore, 48.8% of all IC found were detected in the first round and 65.1% at the initial screening. This table also shows the sensitivity of the screening test. In all cases, the sensitivity of the program (number of cases detected by screening of the total number of tumors found in women screened) lies between 83% and 93%.

**Table 2:** **Incidence rate by age group, screening type in Round 1 and Round 2**

	Women screened N	Interval tumors N	Ratio/10000 (95% IC poisson)	Sensitivity (%)
**50-59 initial**				
**Round 1**	20221	17	8.41 (8.37-8.45)	104/121 (85.95)
**Round 2**	7675	4	5.21 (5.11-5.26)	36/40 (90.0)
**50-59 succesive**				
**Round 2**	14811	9	6.08 (6.04-6.12)	47/56 (83.93)
**60-69 initial**				
**Round 1**	6953	4	5.75 (5.69-5.81)	53/57 (92.98)
**Round 2**	1837	3	16.33 (16.15-16.51)	17/20 (85.00)
**60-69 successive**				
**Round 2**	9449	6	6.35 (6.30-6.40)	42/48 (87.50)

When stratified according to time elapsed between completion of the mammography screening and the diagnostic mammography, we observe that of the initial 43 IC, 10 (23.3%) were detected up to 12 months after the screening mammography and the remaining 33 (76.7%) after this time period (Table 3). Incidence and the proportional incidence rate separated by time elapsed between mammography screening and diagnosis are also shown and stratified similar to in Table 2. During the first year after the screening mammography, the incidence rate is found to be between 0.00 to 5.44 tumors per 10,000 women screened, and the proportional incidence between 0.00% and 23.84%. For IC detected during the second year after the screening mammography, the incidence rate is found to be between 2.88 and 10.89 tumors per 10,000 women screened and the proportional incidence between 12.97% and 47.72%.

**Table 3:** **Incidence rate by age gruopand proportional incidence, screening type and time in Round 1 and Round 2**

Age and type of screening	Time between screening and diagnostic mammography					
	0-11 months			12-24 months		
	Interval tumors N	Ratio/10000 (95% IC poisson)	Proportional incidence (%)	Interval tumors N	Ratio/10000 (95% IC poisson)	Proportional incidence (%)
**50-59 init**						
**Round 1**	4	1.98 (1.96-2.00)	14.38	13	6.43 (6.39-6.46)	46.69
**Round 2**	1	1.30 (1.28-1.33)	9.33	3	3.91 (3.86-3.95)	28.07
**50-59 succ**						
**Round 2**	2	1.35 (1.33-1.37)	9.69	7	4.73 (4.69-4.76)	33.96
**60-69 init**						
**Round 1**	2	2.88 (2.84-2.92)	12.97	2	2.88 (2.84-2.92)	12.97
**Round 2**	1	5.44 (5.34-5.55)	23.84	2	10.89 (10.74- 11.03)	47.72
**60-69 succ**	0	0.00 (0.00-0.00)	0.00	6		27.83
**Round 2**					6.35 (0.61-0.65)	

Finally, Table 4 shows a comparison of the clinicopathological characteristics of the IC with the tumors detected within the program. We found significant differences in the stage, tumor size, the number of positive nodes, histological grade and the progesterone receptor. The IC present a higher proportion of tumors at an advanced stage (14.0% vs. 0.9%), a larger size (5.4% vs. 2.3%), a larger number of positive lymph nodes (13.5% vs. 7.7%), a higher histological grade (37.9% vs. 23.1%) and a higher proportion of cases with negative progesterone receptors (50.0% vs. 26.9%) than tumors detected within the program itself. Although not statistically significant, we detected a higher proportion of triple negative tumors (16.2% vs. 7.5%) and a lower frequency of luminal A (56.8% vs. 69.9%) in the IC than in those from screening.

**Table 4:** **Initial characteristics of interval tumors and tumors detected within PEDBC**

Characteristics	Interval tumors N (%) (n = 43)	Screening tumors N (%) (n = 299)
***Age***		
**50-59**	30 (69.8)	187 (62.5)
**60-69**	13 (30.2)	112 (37.5)
**Total**	**43 (100.0)**	**299 (100.0)**
***Stage*** **		
**0**	4 (9.5)	35 (15.6)
**I**	10 (23.3)	111 (50.0)
**II**	16 (37.2)	59 (26.3)
**III**	6 (14.0)	14 (6.3)
**IV**	6 (14.0)	2 (0.9)
**Total**	**42 (100.0)**	**224 (100.0)**
***Tumor size (cm)*** **		
**≤** **9**	3 (8.1)	60 (27.3)
**10-14**	6 (16.2)	55 (25.0)
**15-19**	6 (16.2)	42 (19.1)
**20-29**	12 (32.4)	44 (20.0)
**30-49**	8 (21.6)	14 (6.4)
**≥50**	2 (5.4)	5 (2.3)
**Total**	**37 (100.0)**	**220 (100.0)**
Lymph nodes**		
**0**	20 (54.1)	150 (71.8)
**1-3**	12 (32.4)	35 (16.7)
**>3**	5 (13.5)	16 (7.7)
**Total**	**37 (100.0)**	**209 (100.0)**
Histological grade**		
**Good**	1 (3.4)	32 (21..8)
**Moderate**	17 (58.6)	81 (55.1)
**Poor**	11 (37.9)	34 (23.1)
**Total**	**29 (100.0)**	**147 (100.0)**
***Histology***		
**Invasive**	38 (90.5)	196 (86.0)
***In situ***	4 (9.5)	32 (14.0)
**Total**	**42 (100.0)**	**228 (100.0)**
***Estrogen receptor***		
**Positive**	30 (75.0)	180 (83.3)
**Negative**	10 (25.0)	36 (16.7)
**Total**	**40 (100.0)**	**216 (100.0)**
Progesterone receptor**		
**Positive**	20 (50.0)	158 (73.1)
**Negative**	20 (50.0)	58 (26.9)
**Total**	**40 (100.0)**	**216 (100.0)**
***HER2***		
**Positive**	10 (27.0)	39 (22.5)
**Negative**	27 (73.0)	134 (77.5)
**Total**	**37 (100.0)**	**173 (100.0)**
***Molecular subtype***		
**Luminal A**	21 (56.8)	121 (69.9)
**Luminal B**	9 (24.3)	28 (16.2)
**HER2-overexpressed**	1 (2.7)	11 (6.4)
**Triple Negative**	6 (16.2)	13 (7.5)
**Total**	**37 (100.0)**	**173 (100.0)**

**Significant differences at 95%.

## Discussion

In our study, we found an IC rate at line with the European guidlines recommendation [6] and lower than the results reported in other studies [15, 19, 20], with a high proportion of cases of true interval cancers (54.5%) and a low proportion of false negatives (13.6%).

Some studies evaluating interval cancers and following the recommendations of the European guidelines have found that about half are true interval cancers, over 20% are false negative [7, 13, 21], and fewer than 20% are occult tumors and minimal-signs cancers [15, 21]. In fact, the false negative is an avoidable interval cancer, as these are tumors that are visible on the mammography but not diagnosed by screening either due to misinterpretation or technical error, and this type of cancer is therefore one that should be found in smaller proportions. Our results are similar to those previously reported by other screening programs (Table 5).

**Table 5:** **Initial characteristics of interval tumors and tumors detected within PEDBC**

	True interval (%)	False negative (%)	Minimal signs (%)	Occult tumors (%)
**Girona**	54.5	13.6	13.6	18.2
**Sabadell-Cerdanyola** [15]	39.5	21.0	26.3	13.2
**West Sussex** [23]	54.3	33.6	-	12.1
**Navarra** [22]	57.7	12.3	15.0	15.0
**Australia** [20]	33.0	41.0	16.0	10.0
**East Anglia** [21]	66.2	17.2	5.4	11.3
**Tarragona** [25]	36.0	24.0	32.0	8.0
**Barcelona** [7]	52.3	20.0	6.2	21.5

As a possible explanatory cause of the low proportion of false negative could be the high immediate recall rate. The immediate recall rate for the Girona program was 16.2% for the first round and 11.9% for the second, which are higher than those recommended in the European guidelines (≤5%) [6]. However, we found that programs with similar proportions of false negative and true interval cancer have a high immediate recall rate too [7, 15, 19, 20, 22–24]. It is certain that an excessively low rate of immediate recall can significantly decrease the sensitivity of screening. There is a clear compromise between the percentage of seconds calls, detections rates and the proportion of IC, and it is necessary to establish the best meeting point for a good sensitivity without unduly compromising specificity [25]. In addition, an increase in the immediate recall rate involve a corresponding decrease in the early-recall rate, and this may reduce pacient anxiety. In the PEDBC the early recall rate has been decreased until 0.6%.

Moreover the protocol classification or the experience of radiologist expert panel could also influence the proportion of false negative and dificult the comparision with others programmes.

On the other hand, the proportional incidence in relation to incidence of breast cancer in the absence of screning programme is an indicator that need to be evaluated in all programmes. In our study we found that in the first year after screening mammography proportional incidence is less than the 30% recommended by European guidelines [6], and the same can be said of the second year, with a proportional incidence of below 50%. These results are better to those observed in other programmes [19, 26, 27]. However, differences in IC definition between studies have to be considered in the interpretation of these results [15].

The IC rate for the PEDBC is within the expected parameters. More in-depth classification of IC and its determinants can contribute to adapting screening practices and improving their effectiveness. It is important for radiologists to know what proportion of true IC, false negatives, minimal signs and occult tumors are diagnosed in order to evaluate and improve their work.

One of the strengths of our study is the use of a population-based registry that has collected data on cancer incidence in the province of Girona since 1980 [28]. Cross-referencing data from the target screening population and all cases of breast cancer has allowed us to identify all probable cases of IC.

However, we should take into account a number of limitations when interpreting the results of our study: 1) the heterogeneity of the different radiological units may affect the ability to detect IC. 2) Partial recovery of the mammograms needed for successful classification. In our study we were only able to recover the two mammograms (screening and diagnosis) necessary for the correct classification of probable interval cancers in 50% of cases as well as in other studies [15]. The main reason for this was the difficulty to obtain the mammography at the clinical record. As missing cases were randomly distributed they probably does not introduce bias. 3) Breast density is a well-known risk factor for breast cancer and particulary interval cancer [29, 30]. Unfortunately, information on breast density is not avaible for the study population.

When we compare our data with those reported in other areas [15, 19, 22–24] it is noticeable that there is a lower proportion of false negatives and a higher proportion of occult tumors on the GHR program. However, the small size of the series must be taken into account.

The comparitive analysis of clinicopathological variables between the IC and cancers diagnosed by screening (Table 4) suggests that IC are more aggressive tumors and are associated with a worse prognosis. These results are similar to those obtained previously in other studies [7]. At the time of diagnosis, interval tumors have a higher proportion of cases with advanced stages and triple negatives. It is widely known that both aspects are associated with a poor prognosis [31]. Tumor size is greater in IC than in those detected by screening. This supports the idea that IC tumors are more aggressive. Also, it is found that most cancers detected by screening are early stage tumors. This reinforces the idea that a diagnostic advance is obtained with the PEDBC.

## Conclusion

This study provides a major evaluation of the PEDBC. Results show that the proportional incidence of IC, as well as the percentage of false nevative IC of the PEDBC is within the limits set by European guidelines. It is important for health professionals to know the true incidence of IC and false negatives in order to improve the effectiveness of the screening program. Furthermore, it has been confirmed that IC display more aggressive clinicopathological characteristics than screen-detected breast cancers.
